# A Call To Action: Developing an Agile Global Regulatory Affairs Workforce for the Future

**DOI:** 10.1007/s43441-025-00877-w

**Published:** 2025-12-05

**Authors:** Lina AlJuburi, Nicole Mahoney, Eddie Reilly, Amy Bertha, David C. Isom, Fabio Bisordi, Virginia Beakes-Read, Michael Garvin, Donna Boyce, Kevin Carl, Sean P. Curtis, Jennifer Dudinak, Carlos O. Garner, Nahid Latif, Sabine Luik, Michelle Rohrer, Katrin Rupalla, Mark Taisey, Raymond Votzmeyer, Max Wegner, Kathy Williams

**Affiliations:** 1 Sanofi, Paris, France; 2 Novartis Pharmaceutical Corporation, East Hannover, NJ USA; 3 Bayer Pharmaceuticals, Wuppertal, Germany; 4 Pfizer, New York, NY USA; 5 Roche/Genentech, Basel, Switzerland; 6 Johnson & Johnson, Titusville, NJ USA; 7 AstraZeneca, Gaithersburg, MD USA; 8 AstraZeneca, Cambridge, UK; 9 Merck & Co., Inc., Rahway, NJ USA; 10 Bristol-Myers Squibb, Madison, NJ USA; 11 Eli Lilly and Company, Indianapolis, IN USA; 12 Takeda Pharmaceuticals, Cambridge, MA USA; 13 GlaxoSmithKline, Munich, Germany; 14 AbbVie, North Chicago, IL USA; 15 Amgen, Thousand Oaks, CA USA

As leaders of biopharmaceutical regulatory affairs organizations, we must learn from the past, thrive in the present and strive for an even better future. We understand the strategic role regulatory affairs professionals play in biopharmaceutical companies, one that uniquely spans the entire continuum of drug development. Our professionals must navigate the increasingly complex regulatory environment, strengthening company engagement with global regulators and other stakeholders to successfully deliver innovative products to patients. Our organizations are only as strong as the people on our teams. As the future of therapeutic development changes in response to technological and scientific advances, we have an obligation to develop, recruit and retain top talent, and build a competency framework to not only meet today’s demands, but to better equip the workforce of the future to improve patients’ access to treatments. There is a great opportunity before us–upskilling our current professionals, attracting individuals with a diversity of expertise into the profession, and setting up the next generation for dynamic and successful careers. This is a call to action for professional societies, academic organizations, regulators, biopharmaceutical companies, and other interested stakeholders to prepare and develop the Global Regulatory Affairs workforce of the future.

## Trends Shaping Drug Development

We are entering a pivotal era in drug development where scientific advances are helping bring new therapeutic modalities, personalized medicines, and novel drug-device combination products to patients more efficiently than ever before. Through digital integration we can generate novel types of evidence and enable more efficient regulatory submissions and reviews. Global regulators are also shifting towards increasing acceptance of innovative trial designs and embracing international collaboration. It is an exciting era for drug development with transformation that requires workforce adaptation.

## Shifting Expectations for Regulatory Affairs Professionals

Understanding the requirements for investigational products, marketing authorizations and compliance have long been the core responsibilities of regulatory affairs professionals. We are relied upon within our organizations as strategic partners for drug development and life-cycle management, helping cross-functional product teams navigate complex regulatory frameworks across different regions, and understand trends in regulatory science and technology. While a deep understanding of regulations and related processes is still a pre-requisite, the drug development landscape now demands professionals who understand the impact that emerging digital technologies and models will have on successful drug development over the next decades; are able to foster cross-functional collaborations within companies; engage externally with stakeholders (e.g. regulators, patients, academia); are aware of how regulatory considerations impact the entire drug lifecycle, including access; can handle risk on multiple dimensions; and, are agile, willing to learn and adapt to emerging trends. We anticipate that over time, companies staffed with such regulatory professionals will gain a competitive edge and better accelerate marketing approvals and patient access to innovative therapies.

## Core Competencies for the Global Regulatory Affairs Workforce of the Future

Regulatory affairs curriculum and training will continue to stand on a solid foundation of knowledge about drug development, medical product and device regulations, and compliance. We have identified the following as key competencies for the future regulatory affairs workforce.

- *Data and digital savvy*—The pharmaceutical industry is investing heavily in new data sources and digital technology, and national health authorities are too. To thrive, the regulatory workforce must have digital literacy skills and proficiency with tools such as dashboards, cloud-based collaborative platforms, generative AI, and AI-enabled workflows. These skills apply not only to increasingly data-driven ways of working, but regulatory affairs professionals also must be confident in understanding how data are acquired, handled, analyzed and incorporated into drug development for regulatory purposes to help product teams meet expectations for marketing approval. They will require a functional knowledge of statistics, data science and epidemiology, to help product teams understand the regulatory expectations associated with using various types of electronic data (e.g., RWD, digital health technology derived data) for evidence generation. Regulatory professionals will also need to understand applicable regulations outside the remit of national health authorities, including those related to data governance, privacy, and cybersecurity, understand how they apply, and when to involve key collaborators (legal, compliance, privacy, etc.).
- *Deeper scientific understanding and broad grasp of the R&D to access continuum*—With the acceleration in new technologies such as gene and cell therapies, a deeper scientific understanding will be important, especially those helping to inform the drug development process. In addition, the once bright lines between research, development and access are blurring to the extent that evidence generation decisions made throughout R&D may have downstream implications not only for marketing approval, but for patient access. Regulatory affairs professionals therefore must have also a holistic understanding of product lifecycles and evidence generation requirements across the entire R&D and access continuum, in response to the use of streamlined regulatory pathways that increase the weight of early research in regulatory decision making, and the greater convergence of clinical research and care.
- *Growth mindset* [1]—The pace of change in our industry and regulatory landscape can best be navigated by a workforce that embraces a growth mindset, meaning a belief that a person’s capacities and talents can be improved over time. Successful regulatory affairs must adopt a self-motivated approach to continuous learning, mentorship, and maximizing skills in leadership and influence within diverse product teams. A growth mindset includes developing leadership skills in interdisciplinary teams, fostering effective scientific collaboration, embracing emerging ‘team science’ methodologies to improve teamwork and research outcomes and developing effective communication skills, including “plain language” approaches to better reach stakeholders with diverse backgrounds.

The authors assessed the current training landscape for regulatory affairs professionals and found that the critical competencies above are generally not being incorporated adequately or systematically into professional society training or collegiate programs. Aside from limited offerings, most available training emphasizes traditional regulatory affairs topics, such as clinical development and applicable regulations for drugs and medical devices. [2–4]

## Recruiting Diverse Expertise

Another avenue to strengthen the workforce is to attract talent with relevant backgrounds (such as data and digital skills) into regulatory affairs. Traditional backgrounds for regulatory affairs professionals include science and biomedical based disciplines such as medicine, pharmacy, nursing, biological sciences, and chemistry, but with the right exposure, individuals with pharmaceutical development experience including statisticians, data scientists could be encouraged to join this dynamic and public health-oriented career path.

## Next Steps

As we focus our efforts, we are posing the following questions:

- Which trends will have the most impact on drug development in the near term? In the long term?
- What should be part of an updated regulatory affairs core curriculum?
- How can we best upskill the current workforce through targeted capacity building?
- How can we attract and develop regulatory affairs professionals from other disciplines (e.g., data scientists, statisticians, clinicians)?
- How can we ensure that regulatory affairs is seen as an attractive profession that draws the best and the brightest?
- Who should we partner with to make this happen?

## Call for Action

We are dedicated to collaboratively addressing these challenges and crafting a comprehensive roadmap for the future of the regulatory affairs workforce. However, this vision cannot be realized in isolation. A multifaceted strategy is essential to upskill the workforce of today and revamp our training. We extend a call to action to professional societies, academic organizations, regulators, Health Technology Assessment (HTA) bodies, biopharmaceutical companies, and other interested stakeholders. Together, we can nurture and expand the global regulatory affairs workforce, ensuring it is well-equipped to navigate the complexities of tomorrow’s healthcare landscape.

Join us in this crucial initiative to shape the future of regulatory affairs and, by extension, the future of global healthcare innovation and patient care.

## Data Availability

No datasets were generated or analysed during the current study.
